# Supplementary material for: MmCMS: mouse models’ consensus molecular subtypes of colorectal cancer
Source: Br J Cancer. 2023 Jan 30;128(7):1333–43. doi: 10.1038/s41416-023-02157-6 (PMC10050155; doi:10.1038/s41416-023-02157-6)
Supplement: Supplementary file 2 — Supplementary File Legends [file 41416_2023_2157_MOESM2_ESM.docx]

**Supplementary excel file 1**

File 1, Sheet 1: The concordance between CMS calls in TCGA data generated by CMScaller (Option-A) and by RF method when set a stringent probability cut off (>0.8).

File 1, Sheet 2: The concordance between CMS calls in TCGA data generated by Option-B and by RF method when set a stringent probability cut off (>0.8).

File 1, Sheet 3: The concordance between CMS calls in TCGA data generated by Option-C and by RF method when set a stringent probability cut off (>0.8).

**Supplementary excel file 2**

File 2, Sheet 1: Characterisation of the mouse samples used in combined cohort.

File 2, Sheet 2: Details of the small independent mouse cohort.

File 2, Sheet 3: Details of the large independent mouse cohort.

**Supplementary excel file 3**

The list of genes used as a template for MmCMS-A classifier after converting the human CMScaller template genes to mouse orthologues.
